# Attitudes toward condom education amongst educators for Deaf and hard-of-hearing adolescents in South Africa

**DOI:** 10.4102/phcfm.v6i1.564

**Published:** 2014-08-13

**Authors:** Sumaya Mall, Leslie Swartz

**Affiliations:** 1 Alan J Flisher Centre for Public Mental Health, Department of Psychology, Stellenbosch University, South Africa

## Abstract

**Background:**

Disabled adolescents are at a critical time in their psychosocial and sexual development.

**Aim:**

This study explores the attitudes of educators working in schools for Deaf and hard-of-hearing pupils in South Africa toward condom education for their pupils.

**Methods:**

We conducted a combination of individual in-depth and joint interviews with a total of 27 participants. The sample comprised educators, school psychologists, school nurses and teaching assistants.

**Results:**

Results showed that educators were aware of the HIV risk for their pupils and reported the risk of sexual abuse or premature sexual activity as being risk factors for HIV infection. None of the schools had a written condom education policy. Whilst some schools were integrating condom education in existing school curricula, others faced moral or religious dilemmas in doing so. There were differences in attitudes, both amongst schools and amongst educators in the same schools.

**Conclusions:**

Given the context of a burgeoning HIV epidemic, it is vital to address adequate condom education in schools.

## Abstrait

**Attitudes des éducateurs d'adolescents sourds et malentendants en Afrique du Sud, envers l’éducation sur l'usage des préservatifs**.

**Contexte:**

Les adolescents handicapés sont à un moment critique de leur développement psychosocial et sexuel.

**Objectif:**

Cette étude examine les attitudes des éducateurs des écoles de Sourds et Malentendants en Afrique du Sud envers l’éducation sur l'usage des préservatifs pour leurs élèves.

**Méthodes:**

Nous avons procédé à une combinaison d'entretiens individuels et en groupe approfondis avec un total de 27 participants. L’échantillon comprenait des éducateurs, des psychologues scolaires, des infirmières scolaires et des maîtres auxiliaires.

**Résultats:**

Les résultats ont montré que les éducateurs étaient au courant des risques de VIH chez leurs élèves et ils ont signalé que le risque d'abus sexuels ou d'activité sexuelle prématurée étaient des facteurs de risque d'infection par le VIH. Aucune des écoles n'avait de politique écrite d’éducation sur l'usage des préservatifs. Bien que certaines écoles aient intégré l’éducation sur l'usage des préservatifs dans leur programme scolaire, d'autres étaient confrontées à des dilemmes moraux ou religieux. On a remarqué qu'il y avait des différences d'attitudes entre les écoles et parmi les éducateurs d'une même école.

**Conclusions:**

Etant donné le contexte d'une épidémie croissante de VIH, il est indispensable d'aborder sérieusement l’éducation sur l'usage des préservatifs dans les écoles.

## Introduction

Despite the fact that people with disabilities may be more vulnerable to HIV than the general population, some HIV services are inaccessible to them.^1^ Accessibility to services is a particular issue for members of the Deaf^1^ community because information is rarely available in accessible formats such as Sign Language.^2^ In addition, particularly in lower-income countries, people with hearing loss are more likely than the rest of the population to face literacy challenges because of their reduced access to adequate schooling. Written materials may also be of little use.^3, 4^ South Africa has the highest number of HIV-positive people of any country, meaning that addressing the issue of prevention is thus a pressing concern for its Deaf community. There is also little information on the needs of this community regarding HIV services.^5, 6^ As part of a larger study,^6^ we report here on data exploring attitudes to sexuality and condom education amongst educators of Deaf and hard-of-hearing pupils in six schools in Gauteng and the Western Cape provinces in South Africa.

## Research methods and design

### Participants and study sites

Having obtained the required ethical and institutional permission, the first author (SM) interviewed educators at six schools for Deaf and hard-of-hearing students in Cape Town and Johannesburg. All the schools catering for this population in Cape Town were selected, as were the two schools in Johannesburg with the longest history of Deaf education. For practical reasons, the two more recently established schools in Johannesburg were not studied. Within the schools, we employed purposive sampling and recruited 27 participants (of whom six were Deaf or hard-of-hearing) across the six schools. We interviewed 19 high school teachers, two school psychologists, one matron or ‘hostel mother’, three teaching assistants and two school nurses through a combination of joint interviews (two participants) and individual in-depth interviews. All had worked at their respective schools for between 10 and 20 years. All hearing participants were interviewed in English or Afrikaans (as per their choice) and Deaf participants were assisted by a South African Sign Language (SASL) interpreter. Interviews were recorded and transcribed by an independent transcription service.

### Measures

We developed an open-ended interview guide by studying key issues from both Deaf studies and behavioural adolescent literature pertaining to sexual behaviour or HIV. Key domains were: challenges facing Deaf and hard-of-hearing youth regarding HIV; schools’ initiatives to address HIV risk and concerns; and schools’ policies on condom usage.

### Data analysis

We used the framework approach, which consists of three phases:^7^ (1) familiarising oneself with the data (i.e. reading the transcriptions, developing codes and assigning data and themes to the codes developed); (2) summarising and synthesising coded data; (3) developing associations or patterns within concepts and themes and ensuring that participants have been presented accurately. Initially, responses were read for emergent themes, which were then coded. Care was taken to ensure the codes captured the respondents’ meaning accurately. A second researcher (LS) coded the interviews independently in order to ensure validity of the categories. We used NVivo 7.0 (QSR International 2008), a qualitative software program, for data management.

### Ethical considerations

As the first author was based at the University of Cape Town (UCT) at the start of the study, ethical approval to conduct the study was provided by the Human Ethics Research Committee of UCT's Faculty of Health Sciences (REC REF 423 2009). The Faculty of Health Sciences Committee at Stellenbosch University later reviewed the application and also provided ethical approval. The research team adhered to all ethical procedures during the research process, including informed consent for all participants where they were assured their responses would remain confidential. They were also assured that they could discontinue their participation in the study at any time.

## Results

### Participants’ views on their pupils’ sexuality

Several participants believed that Deaf children are at risk of sexual abuse. One explained:

‘… people with disabilities, they are at more risk when coming to, to, to sexual activities … they are at more risk [*of sexual abuse*] … they [*the perpetrators*] don't regard them as you know, people, normal people you see, so they take advantage of that …’ (High School teacher 1, Life Orientation, Female)

### Schools’ policies on condom education

None of the schools had a written policy regarding condom usage and educators had different views of the issue. One participant, for example, thought it was important to have condom demonstrations so that Deaf pupils can understand how to use them, but a colleague working in the same school thought that condom demonstrations were problematic and that they could encourage premature sexual activity:

‘So I almost want to say, the thing that's bad, the motivation from the adults [*to*] use a condom … actually encourages, to say … it's all right, … the message that actually comes through is, you can have go ahead and have sex … it's actually a bad message … that is given out to the community …’ (High School teacher 2, Afrikaans, Male)

Another school (although historically Catholic) had a long history of educating pupils about condoms. Two Deaf teachers who had completed their schooling at the school reported that they had received condom education there over 20 years ago:

‘We don't have any nuns here now, when I was young, they gave us sex education, but it did not start from the nuns, they took somebody from outside to present sexuality to us, 22 years ago, they gave us sex education and showed us on a banana how to use a condom.’ (High School teacher 3, Life Orientation, Female)

However, a school nurse mentioned that the school's previous principal (a nun) had not allowed her to distribute condoms to pupils:

‘… [*u*]ntil the end of last year, we had a, a nun that was our principal … I was given a donation of condoms … and I wanted to hand them out and she wouldn't allow me to … we have a volunteer – she's been doing the sex education with the children. She would hand out condoms in her classroom, so I gave her the box, so the condoms did eventually get to the kids.’ (School Nurse 1, Female)

Although the principal at another Catholic school reported that staff were under pressure from the nuns not to have condom demonstrations at school, four participants working at that school said that demonstrations did, in fact, occur. The principal also conceded the reality of early sexual risk behaviour amongst pupils and the necessity of condoms:

‘At the very least [*I tell the pupils*] use condoms, which I know as a Catholic I shouldn't promote, but I mean if a child is already sexually active …’ (School Principal 1, Female)

The principal of another historically Catholic school was adamant that condom demonstrations were not allowed at her school:

‘It's [*condom usage*] not encouraged and I respect, I am not a Catholic but I am the principal of this school and I had, I have decided to come and work in this school and … I feel it's right that I uphold their ethos. If I feel, if I can't do that then … I should terminate my service because I have applied to be in this school.’ (School Principal 2, Female)

However, one of her staff members, a teacher, explained that there may be room for adaptation of certain values:

‘The owners of the school are Catholic sisters … but now I've put a poster up there with A, B, C [*abstain, be faithful, condomise*] … and nobody has taken down you know, take them down … the only thing they would not allow is the condom dispensers.’ (High School teacher 4, Life Orientation, Female)

## Discussion

Our findings reveal a mixed picture. Whilst one school had no limitations regarding condom education, religious and moral issues influenced two of the other schools’ condom education programmes. One Catholic school had successfully managed to reconcile religious belief with HIV prevention needs. Whilst educators in the other traditionally Catholic schools were making efforts to do the same, they were not always supported by their principals, who believed that a Catholic stance on condom usage should be upheld.

It may be chance (for example, dependant on who a child has as a teacher) that decides what kind of sex education (if any) a child in a school for Deaf and hard-of-hearing pupils receives and whether or not the child learns about condom use. For some educators, there seems to be a clash between their religious values and the threat of an emerging HIV epidemic. This finding resonates with that of Rohleder and Swartz,^8^ a study that found that carers feared that providing HIV prevention information to disabled people would place them at risk of sexual abuse. Rohleder^9^ found that staff at disability organisations sometimes viewed condom education for disabled people as being immoral; their views were grounded in religious beliefs. Given the vulnerability of Deaf and hard-of-hearing adolescents to HIV infection, this issue is not simply one of values. Providing sexuality education to pupils who are Deaf or hard-of-hearing poses challenges at the best of times, because of the communication barriers, but as far as we are aware, the question of the role of religious beliefs and values has not (until now) been explored sufficiently. As quoted above, one of the participants says, ‘I have decided to come and work in this school’ and, therefore, out of respect for the school, believes that it is incumbent on her to operate in keeping with the values she sees as being central to the school. This is an admirable stance and resonates with attitudes toward contraception within many religious bodies and organisations.^10^

## Conclusion

The parents of the pupils have had no such choice in terms of the values of the school – either the pupils attend this school or they do not receive education. This may well have an impact on their future health. This situation, it is important to recognise, is a product not of the views of the educators themselves (they are entitled to their own beliefs regarding sexuality education) but of the social context in which education for Deaf pupils (and indeed for many other disabled children) continues to be coupled with religious institutions. Many within the schools, as we have shown, are doing all they can to provide sexuality education. A broader engagement, balancing the rights of religious schools (and educators) to their beliefs against the rights of the pupils to have access to sexuality and health promotion, needs to be undertaken.

## Footnotes

1 We use the capital letter ‘D’ to denote that Deafness is a cultural identity that refers to individuals with hearing loss who communicate in Sign Language and are members of a Deaf community
